# Function of basal ganglia in bridging cognitive and motor modules to perform an action

**DOI:** 10.3389/fnins.2014.00187

**Published:** 2014-07-08

**Authors:** Atsuko Nagano-Saito, Kristina Martinu, Oury Monchi

**Affiliations:** ^1^Centre de Recherche, Institut Universitaire de Gériatrie de MontréalMontréal, QC, Canada; ^2^Department of Radiology, Université de MontréalMontréal, QC, Canada

**Keywords:** basal ganglia, dopamine, fMRI, cross-network synchrony, Parkinson's disease

## Abstract

The basal ganglia (BG) are thought to be involved in the integration of multiple sources of information, and their dysfunction can lead to disorders such as Parkinson's disease (PD). PD patients show motor and cognitive dysfunction with specific impairments in the internal generation of motor actions and executive deficits, respectively. The role of the BG, then, would be to integrate information from several sources in order to make a decision on a resulting action adequate for the required task. Reanalyzing the data set from our previous study (Martinu et al., 2012), we investigated this hypothesis by applying a graph theory method to a series of fMRI data during the performance of self-initiated (SI) finger movement tasks obtained in healthy volunteers (HV) and early stage PD patients. Dorsally, connectivity strength between the medial prefrontal areas (mPFC) and cortical regions including the primary motor area (M1), the extrastriate visual cortex, and the associative cortex, was reduced in the PD patients. The connectivity strengths were positively correlated to activity in the striatum in both groups. Ventrally, all connectivity between the striatum, the thalamus, and the extrastriate visual cortex decreased in strength in the PD, as did the connectivity between the striatum and the ventrolateral PFC (VLPFC). Individual response time (RT) was negatively correlated to connectivity strength between the dorsolateral PFC (DLPFC) and the striatum and positively correlated to connectivity between the VLPFC and the striatum in the HV. These results indicate that the BG, with the mPFC and thalamus, are involved in integrating multiple sources of information from areas such as DLPFC, and VLPFC, connecting to M1, thereby determining a network that leads to the adequate decision and performance of the resulting action.

## Introduction

The basal ganglia (BG), mainly consisting of the striatum, the globus pallidus, the substantia nigra, and the subhalamic nucleus, are involved in a variety of functions. The striatum [caudate, putamen, and ventral striatum (VS)] is the main input of the BG, and the thalamus connects the BG to the cortex through cortico-BG-thalamo-cortical loops and brainstem-thalamo-BG-brainstem loops (Alexander et al., 1986; Albin et al., 1989; McHaffie et al., 2005). The striatum receives extensive glutamatergic afferents from a wide variety of cortical regions (Parent and Hazrati, 1995). The dopaminergic projections from the midbrain to the striatum are a crucial modulator of striatal processing of glutamatergic cortical and thalamic signals on the striatum principal neurons (Surmeier et al., 2009). Patients with Parkinson's disease (PD), characterized by motor and cognitive dysfunctions, show significant reduction of the dopaminergic projections to the striatum (Samii et al., 2004). Patients generally display difficulties in performing internally generated movements (Georgiou et al., 1993), and cognitive deficits often consist of decreases in executive functions (Litvan et al., 1991). Internal generation of movement and executive functions both require decision-making processes in order to select an action among several alternative possibilities for the task at hand. The BG, then, mostly modulated by dopaminergic projections, seem to have an important role in the mediation of cognitive and motor modules to generate an appropriate decision on a resulting action for the task being performed.

In the context of brain networks, it is not clear how BG neuro-modulation affects decision making, and how it is impaired by dopaminergic dysfunction. Previous studies indicate that executive deficits in PD are associated with dysfunction of the caudate nucleus in PD patients without dementia (Lewis et al., 2003; Monchi et al., 2004, 2007). This suggests that dopamine is involved in the transfer of information first processed in cognitive brain networks, toward motor-related networks, sequentially. Furthermore, PD patients with mild cognitive impairment (MCI), compared with non-MCI patients, showed dysfunction of the caudate nucleus, along with a stronger reduction of activity in the motor cortex, but without any differences in motor-ability (Nagano-Saito et al., 2013). As of now, however, it is not clear how the BG are organized in order to effectively combine cognitive and motor modules to conduct a series of tasks. While performing the Wisconsin Card Sorting Task (WCST), we observed increases in functional connectivity between the frontal regions [including the dorsolateral prefrontal cortex (DLPFC), the ventrolateral prefrontal cortex (VLPFC), and the pre-supplementary motor area (pre-SMA)], and the putamen in healthy volunteers (HV). This effect was diminished with transient dopamine depletion (Nagano-Saito et al., 2008). Moreover, the strength of the connectivity between the DLPFC and the striatum could estimate individual response times (RT) (Nagano-Saito et al., 2008). Another study with self-initiated (SI) movements showed increased functional connectivity between cortical areas (pre-SMA and the primary motor area) and the putamen in HV; this was diminished in PD patients (Wu et al., 2011). Since the DLPFC and the pre-SMA are components of the cognitive network (Alexander et al., 1986; Albin et al., 1989; Picard and Strick, 2001), the resulting fMRI functional connectivity may reflect the combination of both the cognitive and motor modules.

SI finger movements are simple, but require a certain level of decision-making, such as selecting one action among several alternative possibilities. SI movements have been shown to recruit a larger number of brain activations, including the DLPFC, VLPFC, medial prefrontal cortex (mPFC), striatum, and thalamus, compared with single-button presses (Francois-Brosseau et al., 2009; Martinu et al., 2012). These activations were decreased in PD patients (Martinu et al., 2012). PD patients are impaired in the internal generation of movement (Georgiou et al., 1993; Kiesel et al., 2010), plausibly linking the impairment of movement prediction for task preparation (Werheid et al., 2007). The motor dysfunction in PD patients may be related with the impairment in decision-making processes, like predicting possible actions. Based on animal single cell recordings, decision making properties seem to be expressed by the same cells as sensorimotor processes, joining what should be undertaken by cognitive processes with movement execution, rather than showing localization within particular higher cognitive centers in the brain (Cisek and Kalaska, 2010). In humans, even simple perceptual decisions emerge from recurrent and flexible interactions between widely distributed regions of the cerebral cortex, showing an oscillatory interaction between the regions (Siegel et al., 2011). Considering these finding, the oscillation of interaction (e.g., between the cognitive and motor modules) during decision-making processes required for the SI task could be operated by the BG system.

The application of graph theory methods to brain imaging data is a simple and powerful mathematical framework for the characterization of topological features of brain networks (Bullmore and Sporns, 2009; He and Evans, 2010). The methods enable us to investigate the structural features in the brain, including both the local-, and global-level connectivity. In graph theory, a network consists of nodes and edges (connections between any two nodes). The network wiring cost is defined as the ratio of actual connections to the maximum number of possible connections, and the topological features of networks, such as global, local, and cost efficiencies, are known to change as the function of wiring costs change (Latora and Marchiori, 2001; Bullmore and Sporns, 2009; He and Evans, 2010). The global efficiency is an index of inverse path length, defined by an average minimum number of connections that link any two nodes of the network, and indicates the efficiency of information transfer among different brain regions (Achard et al., 2006; He and Evans, 2010). The local efficiency is defined as an average of the clustering coefficients over all nodes in the network, and a measure of local clustering and fault tolerance (Achard et al., 2006; He and Evans, 2010). Functional MRI and MEG resting-state and task-based studies with this method have shown that the human brain has small-world network properties with a certain range of network wiring cost (Achard et al., 2006; Bassett and Bullmore, 2006; Achard and Bullmore, 2007; Bassett et al., 2011; Kitzbichler et al., 2011; Carbonell et al., 2014). This method also unveiled that PD patients decreased local connectivity function in the mPFC, prefrontal cortex and striatum, and globally measured network efficiency indicated lowered information flow in the brain (Wu et al., 2009; Cao et al., 2011; Skidmore et al., 2011; Baradaran et al., 2013; Gottlich et al., 2013).

A small-world network consists of nodes that are not necessarily neighbors, but can be reached by a small number of steps, displaying an enhanced signal-propagation speed (Watts and Strogatz, 1998). The networks with small-world network properties show greater local efficiency than a random network, and less global efficiency than a random network but still greater than a regular lattice network (Achard et al., 2006; Achard and Bullmore, 2007; Skidmore et al., 2011; Carbonell et al., 2014). The small-world network property is considered as the outcome of a selection by competitive criteria: minimization of wiring cost vs. maximization of efficiency of information transfer (Bassett and Bullmore, 2006; Kitzbichler et al., 2011). Cost efficiency is defined as “global efficiency – cost,” and it is assumed that the brain operates optimally with the maximum cost efficiency, maximizing information transfer (Sporns et al., 2000; Achard et al., 2006; Achard and Bullmore, 2007; Bassett et al., 2009).

Intrinsic patterns of functional connectivity of the human brain have been shown in the visual, auditory, motor, task-control, and default mode networks (Biswal et al., 1995; Greicius et al., 2003; Fox et al., 2005; Power et al., 2011), corresponding to relatively high local efficiency (Kitzbichler et al., 2011). The small-world network property hypothesis suggests rapid cross-network synchronization, which plays a crucial role in information processing (Lago-Fernandez et al., 2000; Masuda and Aihara, 2004; Bassett et al., 2011). Patterns of the brain network are rapidly modulated by task complexity, showing cross-network synchronization during more complex tasks, possibly supported by relatively high global efficiency of the brain (Kitzbichler et al., 2011). Thus, considering small-world network properties while applying graph theory methods during a task would enable us to study a condition of the global brain connectivity, crucial for the performance of a given task. The graph theory approach also helps in investigating the local connectivity one by one, such as between nodes belonging to the cognitive and motor networks, and between nodes belonging to the cortical and subcortical areas.

In the present study, we wanted to explore the hypothesis that the BG play an important role in the integration of cognitive and motor networks in order to proactively perform tasks. We used a previous fMRI data set from HV and PD patients performing SI finger movements (Martinu et al., 2012). More specifically, we first investigated economical connectivity considering the maximum cost efficiency with a feature of small-world network during the SI finger movement task in the HV by applying the graph theory methods with a cost-threshold approach. Here, the individual networks were structured with fixed costs, rather than with fixed correlation threshold (the minimal correlation ratio). The cost-threshold approach was applied in order to consider the maximum cost efficiency, and because this approach is more relevant in the analysis of connectivity “patterns” between different populations (HV vs. PD). We then compared this pattern with PD patients' connectivity. In addition, we investigated whether the activity of the striatum in relation to cortico-cortical and cortico-striatal connectivity could estimate individual behavior.

## Materials and methods

### Participants

Fourteen right-handed HV (mean age 61.74 ± 6.62 years, six males) and 12 right-handed patients at the early and moderate stages of idiopathic non-demented PD (mean age 62.89 ± 6.70 years, six males) were recruited (for detailed patient information, see Martinu et al., 2012). All patients were diagnosed by a movement disorders neurologist and met the United Kingdom Brain Bank criteria for idiopathic PD (Hughes et al., 1992). All patients were in Hoehn-Yahr stage I or II (Hoehn and Yahr, 1967). At the time of fMRI scanning, patients were asked to withdraw from all antiparkinsonian medications at least 12 h prior to the appointment. All subjects gave written informed consent to the protocol which was reviewed and approved by the research ethics committee of the Regroupement Neuroimagerie Quebec (CMER-RNQ) at the Institut Universitaire de Gériatrie de Montréal, following the guidelines of the Tri-Council Policy Statement of Canada, the civil code of Quebec, the Declaration of Helsinki, and the code of Nuremberg. All the participants who declined to participate or did not participate were not disadvantaged in any other way by not participating in the study. None of the participants had a compromised ability to consent on their own.

### Behavioral tasks

Using both their right and left hands separately, participants performed a SI random movement condition, an externally-triggered (ET) follow condition, and a single-button repeat condition as a control (CTL). Five blue squares were displayed, each square corresponding to a button on the response box; all fingers were used except for the little finger, as it was considered too difficult for patients. For the SI condition, all four squares would turn green, and the subject had to form his/her own sequence of button-presses. As a feedback, the buttons pressed made the green squares turn yellow immediately, after which the next button was ready to be selected. Pressing the same button twice in a row was considered to be an error, indicated by the equivalent square turning red. We also asked the participants to refrain from using common sequences, such as 1-2-3-4 and 4-3-2-1, or repeating sequences, such as 4-2-3-1-4-2-3-1… Participants' performance was monitored to insure they kept their movement sequences as random as possible. During the ET condition, the subject had to follow the sequence as the blue squares alternately turned green. During the control condition, one square switched from blue to green, indicating that the corresponding button should be pressed. All the conditions with left and right hands were performed in every run of the fMRI scans, and the task included a total of 20 button presses per condition. For detailed task information, see (Francois-Brosseau et al., 2009; Martinu et al., 2012). The data during SI were used for this study.

### Behavior measurement

Individual averages of the RT during each task (SI, ET, and CTL) were calculated. The RT was defined as the time between the beginning of a button press to the beginning of the following button press. Data for the left and right hands were combined for the purpose of the present study. The averaged RT during SI movements was used as an index of performance level of subjects' behavior.

### fMRI scanning

Subjects were scanned at the Unité de Neuroimagerie Fonctionnelle (UNF) of the Center de Recherche de l'Institut Universitaire de Gériatrie de Montréal (CRIUGM) using a 3T TIM Siemens Magnetom MRI scanner. Each scanning session began with a high- resolution, T1-weighted, three-dimensional volume acquisition for anatomical localization (1 mm^3^ voxel size), followed by four sets of echoplanar T2^*^-weighted image acquisitions with blood oxygenation level-dependent (BOLD) contrast (echo time, 30 ms; flip angle, 90°). Each run consisted of 146 frames of 43 slices (matrix size, 128 × 128 pixels; voxel size, 3.34 × 3.34 × 3 mm^3^, TR: 3.5 s). The number of total volume for each subject was 584.

### fMRI preprocessing

We applied the NIAK preprocessing pipeline on fMRI data sets (Bellec et al., 2012). First, slice timing correction was performed with spline interpolation. After motion correction, slow time drift was removed from the BOLD time series with a high-pass filter of 0.015 Hz. Images were then transformed into ICBM 152 space, and spatially blurred with a 4 mm full width half-maximum isotropic Gaussian kernel.

### Regions of interest (ROI) and time series extraction

We generated task-related networks on specific tasks by selecting 62 regions of interest (ROI) as nodes. Fifty-eight ROIs were taken from the activation maps acquired by comparison between events (Martinu et al., 2012) in the HV with a threshold of *t* = 3.5. They included 44 ROIs generated from the SI minus CTL contrast for left and right-hand movements combined, and 14 ROIs generated from the comparison between the right and left hands in the CTL condition. These ROIs included three of the putamen and three of the thalamus. We also added four ROIs, which were located in the caudate nucleus and the VS, bilaterally. All the ROIs are shown in the Table 1, with additional information for activation pattern. In graph theory, these ROIs are considered as nodes of the brain network, and the connections between every two ROIs are considered as edges. The ROIs consisted of small cubes (total of 27 voxels) centered on each peak in ICBM 152 space. Time series for the entire task duration (34 min, 584 volumes) were extracted for each ROI for each subject and session.

**Table 1:** **Regions of interest in the brain (in ICBM 152 space)**.

**ROI**	***x***	***y***	***z***
**SI > ET, SI > CON**			
SMA1	−4	−2	68
PreSMA1	6	16	46
VLPFC1	−50	4	4
VLPFC2	44	14	2
VLPFC3	56	14	0
DLPFC1	34	40	36
APFC	40	46	2
INS1	−32	18	4
INS2	30	20	6
Thal1	−14	−18	6
Thal2	14	−16	8
LOA1	50	−54	−6
**SI > ET, SI > CON, ET > CON**			
PMd1	−20	−2	62
PMd2	24	−4	58
IPL1	36	−44	50
Put1	−24	2	4
Put2	24	4	8
**SI > CON, ET > CON**			
PreSMA2	−6	6	48
PreSMA3	6	4	52
PMd3	−32	−12	56
PMd4	38	−10	62
IPL2	−48	−42	54
IPL3	−32	−50	52
IPL4	46	−34	40
DLPFC2	−56	4	36
DLPFC3	52	6	36
IPL5	−28	−60	58
IPL6	18	−66	58
IPL7	−16	−72	56
SOG1	30	−68	32
SOG2	−26	−74	24
SOG3	32	−74	26
SOG4	−28	−82	22
MOG1	−32	−86	12
MOG2	32	−80	6
MOG3	40	−74	4
LG1	−16	−86	4
LG2	18	−90	2
LG3	−10	−84	−10
LG4	14	−84	−12
FG1	−24	−76	−14
FG2	22	−76	−14
LOA2	−48	−68	−8
Cereb1	6	−70	−20
**MTR LEFT VS. RIGHT**			
M1-1	−40	−20	52
Cereb2	−26	−62	−24
Cereb3	28	−58	−24
Cereb4	−28	−66	−52
Cereb5	28	−66	−52
M1-2	40	−22	54
SMA2	−6	−20	50
SMA3	8	−24	50
InsPost1	−36	−32	22
InsPost2	42	−22	20
Cereb6	−18	−58	−20
Cereb7	20	−52	−20
Thal3	16	−20	2
PutPost	32	−12	2
**ETC**			
Caudate1	−14	12	12
Caudate2	14	12	12
VS1	−10	14	−6
VS2	10	14	−6

Abbreviations: SMA, supplemental motor area; PreSMA, pre-supplemental motor area; VLPFC, ventrolateral prefrontal cortex; APFC, anterior prefrontal cortex; INS, insula; Thal, thalamus; LOA, lateral occipital area; PMd, dorsal premotor cortex; IPL, intreaparietal lobe; DLPFC, dorsolateral prefrontal cortex; SOG, superior occipital gyrus; MOG, middle occipital gyrus; LG, lingual gyrus; FG, fusiform gyrus; M1, primary motor area; Cereb, cerebellum; Put, putamen; PutPost, posterior putamen; InsPost, posterior part of Insula; VS, ventral striatum.

### Normalizing and dividing time series

Each time series of single runs from the 62 ROIs were globally normalized using the average and standard deviation of all the extracted BOLD intensities (146 volumes × 62 ROIs). The normalized time series were then divided into three, according to the task period (SI, ET, and CTL). Because of the hemodynamic delay inherent in BOLD signal after stimuli (Buxton and Frank, 1997), the first 10 s of each task period were removed. Finally, all the processed time series of the 4 runs during the SI were combined, individually, on which the network analysis was conducted.

### Averaged BOLD signals through time series, and activation of SI-CTL in subcortical regions

The normalized time series of 10 subcortical ROIs (caudate, putamen, VS, and the thalamus) during the SI were averaged for each subject. This measurement was considered as an index of regional activity during the tasks. For the PD group, from the activation maps acquired by comparison of SI – CTL (Martinu et al., 2012), individual *t*-values of brain activity was calculated. In the previous study, activation in the striatum and thalamus was reduced in the PD patients (Martinu et al., 2012). Therefore, the individual *t*-value in the subcortical ROIs was considered as an index of capacity/incapacity of the dopaminergic function in the PD patients.

### Network analysis

#### Small-world property

Using each individual data set of the normalized BOLD signal time course, Pearson's correlation coefficients was calculated between each pair the 62 ROIs, resulting in a symmetric 62 × 62 correlation matrix. Applying the cost-threshold approach on the 62 × 62 correlation matrix, we first checked the cost, which indicated small-world network property, showing global efficiency is less than random network but greater than a regular lattice network, and local efficiency is more than random network (Achard et al., 2006; Achard and Bullmore, 2007; Skidmore et al., 2011; Carbonell et al., 2014), in the HV and PD patients, respectively. Cost efficiency (global efficiency – cost) was calculated to see the economical cost, while maximizing the cost efficiency during the task, the HV and PD patients, respectively.

#### Network structure—pattern of brain network in HV and PD

The change of the connectivity based on cost was investigated by visual inspection. Based on the results of small-world property, cost efficiency, and visual inspection, we selected the cost of 0.28 to determine the individual networks for the SI task, in the HV and PD, respectively. The edges included in more than half for each group were considered for the further analysis.

#### Group comparison between HV and PD

To investigate group differences in overall connectivity, strength of the connectivity of all linkages (edges) during the SI was compared between groups. For this, Fischer's Z transformation was applied to each individual correlation matrix. Then, inter-group comparisons with *t*-test between the HV and PD groups were performed. Significance was set at *p* < 0.05 (uncorrected). Combined with the results of the correlation analysis mentioned below, we explored the edges affected by the disease.

#### Correlation analysis between correlation ratio of edges and activation of subcortical regions in HV and PD

We performed a correlation analysis between the correlation ratio after Fisher's transformation of the edges above, and the activity in the subcortical regions, with SI condition. This analysis was done, in the HV and PD, respectively. The average BOLD signal through time series were used for the index of subcortical activity. In PD, t-activation calculated by SI – CTL conditions was also used for the analysis, as well as the index of incapacity of the dopaminergic function in PD patients. We reported the results with the significant threshold set at *p* < 0.05 (uncorrected).

#### Relationship between correlation ratio with subcortical connections and task performance in HV and PD

Based on our previous study (Nagano-Saito et al., 2008), we assumed that stronger connectivity between the DLPFC and the striatum would correspond with the faster RT. We were also interested in whether any other correlation between other nodes and the subcortical regions, and weakened in PD, could explain the RT. We therefore calculated individual correlation ratio between these 32 regions and the striatum and the thalamus, then performed a correlation between the correlation ratio after Fisher's transformation and the RT(SI) in the HV and PD patients, respectively. The significant threshold was set at *p* < 0.05 (uncorrected, for DLPFC, and FDR-corrected for others).

#### Difference of path length from the primary motor area between groups

The path length is the number of edges that the path uses. We assumed that the path length from primary motor area (M1) to the striatum, mPFC, and associative cortex is longer in PD patients compared with HV. To confirm this, we calculated the shortest path length from the left and right primary motor area to the other nodes, individually, and compared HV and PD. Wilcoxon rank sum test was used. We reported the results with the significant threshold set at *p* < 0.05 (uncorrected).

## Results

### Behavior

RT for the SI, ET, and CTL were 790.6 ± 208.3, 1045.5 ± 172.8, 826.6 ± 174.4 ms respectively in HV, and 888.67 ± 161.4, 1152.6 ± 208.7, 733.2 ± 112.3 ms respectively in PD patients. A mixed-design repeated measures ANOVA (task × group) showed a significant main effect of the task (*F* = 44.956; *p* < 0.001), but not group (*F* = 0.108; *p* = 0.745). There was also a significant interaction of task × group (*F* = 5.178; *p* = 0.016), showing that the RT for SI was shorter than for CTL in HV, and RT for CTL was shorter than SI in PD patients.

### Network analysis

#### Small-world network property

**Figure 1:**
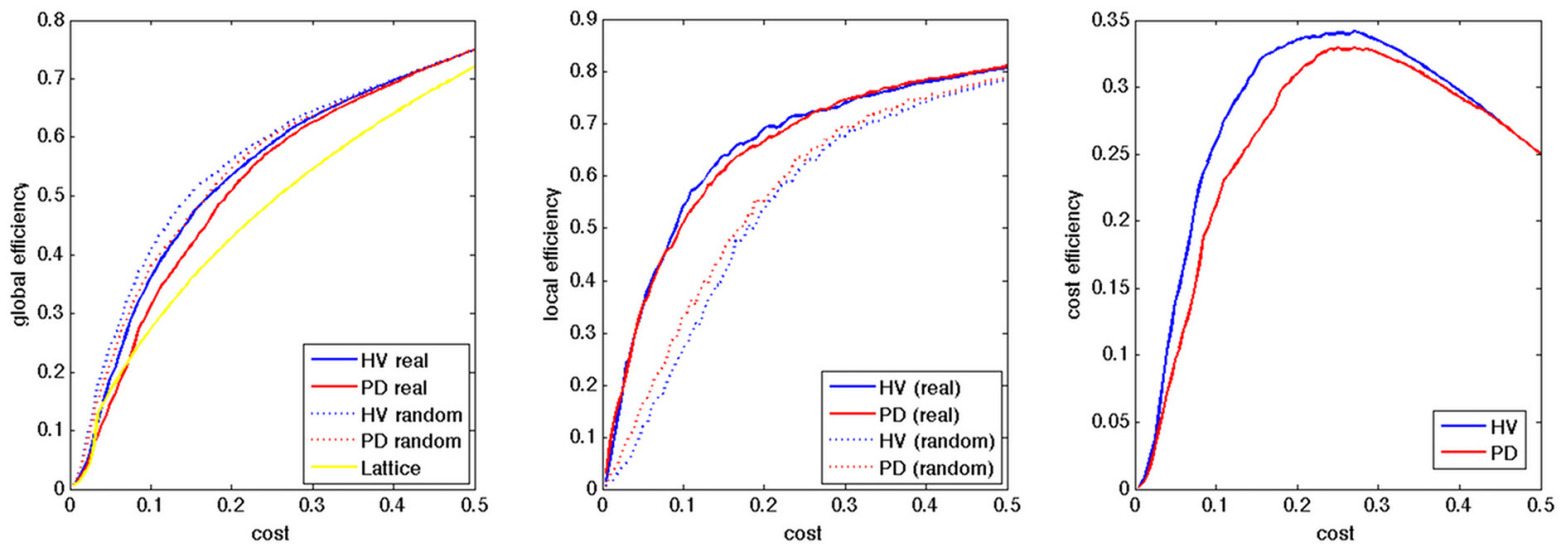
**Group average of global efficiency, local efficiency, and cost efficiency in HV and PD**. The X axis indicates the wiring cost.

All the networks in the HV and PD showed small-world network property in the range of the cost of [0.045–0.460], and of [0.080–0.485], respectively, with less global efficiency and more local efficiency, compared to random networks, and more global efficiency compared to lattice networks (Figure 1). After smoothing the group averaged data using matlab toolbox (preserving the shape option), we obtained the peaks of the cost efficiency at the cost of 0.271 and 0.284, for the HV and PD groups, respectively, and we selected the cost of 0.28 for further analyses.

#### Network structure—pattern of brain network in HV and PD

**Figure 2:**
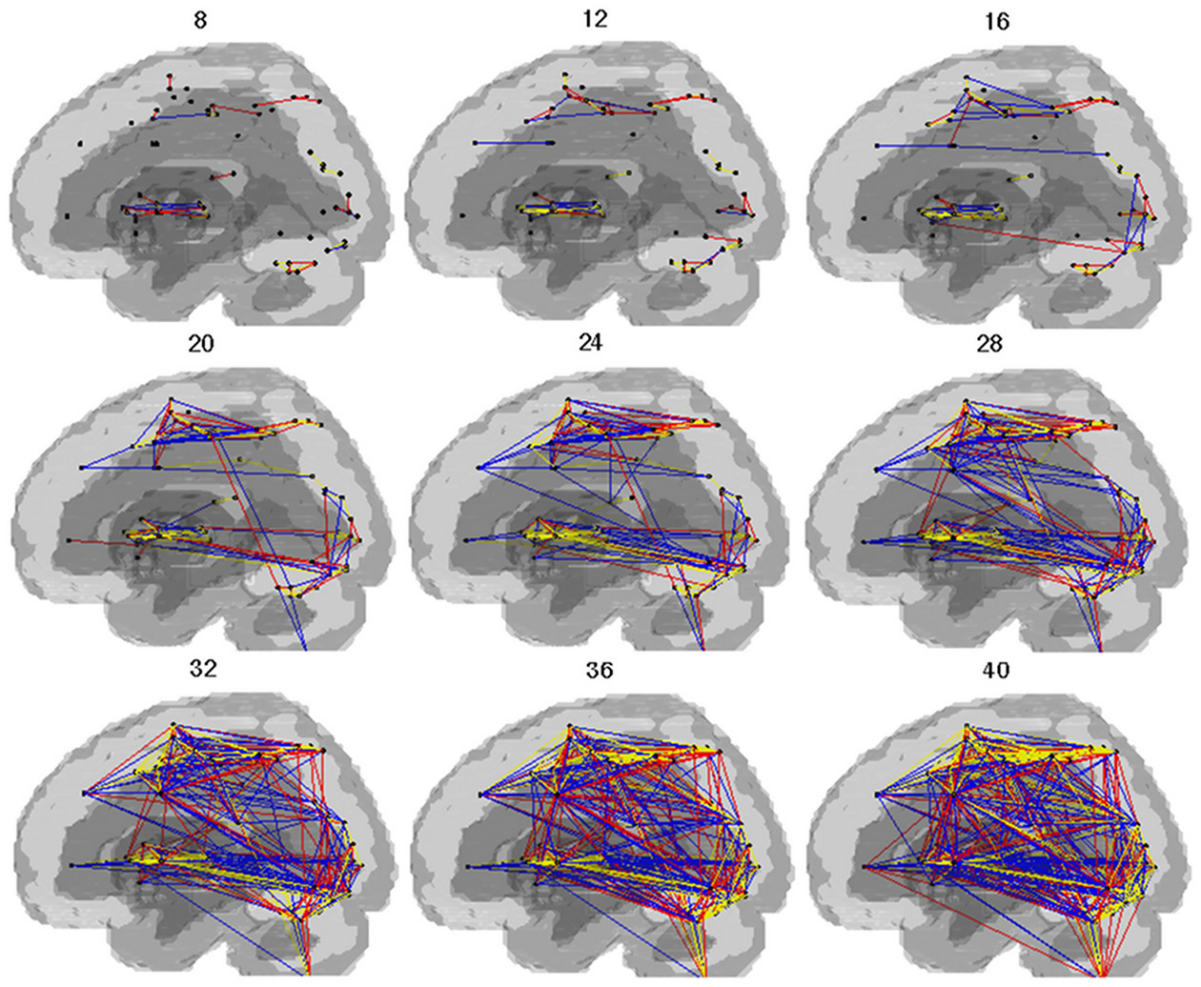
**Pattern of networks in HV and PD**. Lines indicate the edges for which more than half of the subjects in the group showed connectivity with costs. The numbers in the figure indicate the wired cost (%). Yellow lines indicate the edges observed in both the HV and PD. Blue lines indicate the edges observed in the HV. Red lines indicate the edges observed in the PD.

Cost-dependent patterns of brain network during SI are shown in the Figure 2. In both groups, connectivity in the dorsal and ventral regions of the brain was observed above the cost of 0.20. With the individual cost of 0.28, the mean correlation threshold (the minimal correlation ratio) was 0.261 ± 0.0064 in HV and 0.245 ± 0.0582 in PD group, and the total connectivity with our criteria was 12.6% in HV, and 10.9% in the PD group. The connectivity with striatum and the thalamus (excluding inter-connections) were confined to the extrastriate visual cortex and the VLPFC and insula. All the connectivities in the HV and PD, respectively, with our criteria are shown in the Complementary Data (C-Table 1).

#### Group comparison between HV and PD

All results are shown in the Tables 2, 3. The PD patients show weakened connectivity between the mPFC and other cortical areas, including the primary motor area (M1), the extrastriate visual cortex and the associative cortex. Simultaneously, however, PD patients display a few regions of increased connectivity with the mPFC. In the PD patients, the striatum and the thalamus have reduced connectivity with the extrastriate visual cortex, and the connectivity between the VLPFC and the striatum, between the M1 and the posterior insula, as well as with the cerebellum (Cereb) for the left and right sides, respectively, was weakened. Inter connectivity between the left and right DLPFC and within the subcortical regions were also reduced in the PD patients. However, the connectivity between the bilateral VS and intra extrastriate visual cortex was stronger in the PD.

**Table 2:** **Differences in edge strength connectivity between HV and PD during the finger movement tasks (HV > PD)**.

**Edges (HV > PD) (OFF)**		***p*-value**	**Subcortical activity**	
			**HV**	**PD**
PreSMA1	DLPFC1	0.0463	^*^	^*^
PreSMA1	IPL2	0.0232	^*^	^*^
PreSMA1	SMA3	0.0003	^*^	^*^
PreSMA1	InsPost1	0.0023	^*^	^*^
PreSMA1	SOG4	0.0342		^*^
PreSMA1	SMA2	0.0434	^*^	
PreSMA1	SOG1	0.0263	^*^	^*^
PreSMA2	M1-1	0.0094		^*^
PreSMA2	SMA2	0.0392	^*^	^*^
PreSMA3	PMd2	0.0246		^*^
SMA1	M1-1	0.022		^*^
SMA1	IPL5	0.0444		^*^
SMA2	DLPFC2	0.0415		^*^
DLPFC1	DLPFC2	0.0249	^*^	
DLPFC3	SOG1	0.002	^*^	^*^
DLPFC3	SOG3	0.0331		^*^
M1-1	InsPost1	0.0319		
M1-2	Cereb6	0.0489		
VLPFC3	Put1	0.0362		^*^
Put2	Thal1	0.0236	^*^	
Put2	Thal2	0.0131	^*^	^*^
PutPost	VS1	0.0136		^*^
VS1	LG3	0.0022	^*^	
Thal1	LG2	0.0079	^*^	^*^
Thal1	FG1	0.0147		
Thal3	MOG3	0.0226	^*^	
Thal3	FG1	0.0054		

Edges whose connectivity strength was positively correlated to subcortical (striatum and/or thalamus) activation are marked by an asterisk.

**Table 3:** **Difference in edge strength connectivity between HV and PD during the finger movement tasks (PD > HV)**.

**Edges (HV < PD)**		***p*-value**
PreSMA3	LG4	0.0489
SMA2	Cereb7	0.0088
SMA3	IPL1	0.0388
VS1	VS2	0.0077
MOG1	LG1	0.0229
MOG1	LG2	0.0448

#### Correlation analysis between correlation ratio of edges and activation of subcortical regions in HV and PD

**Figure 3:**
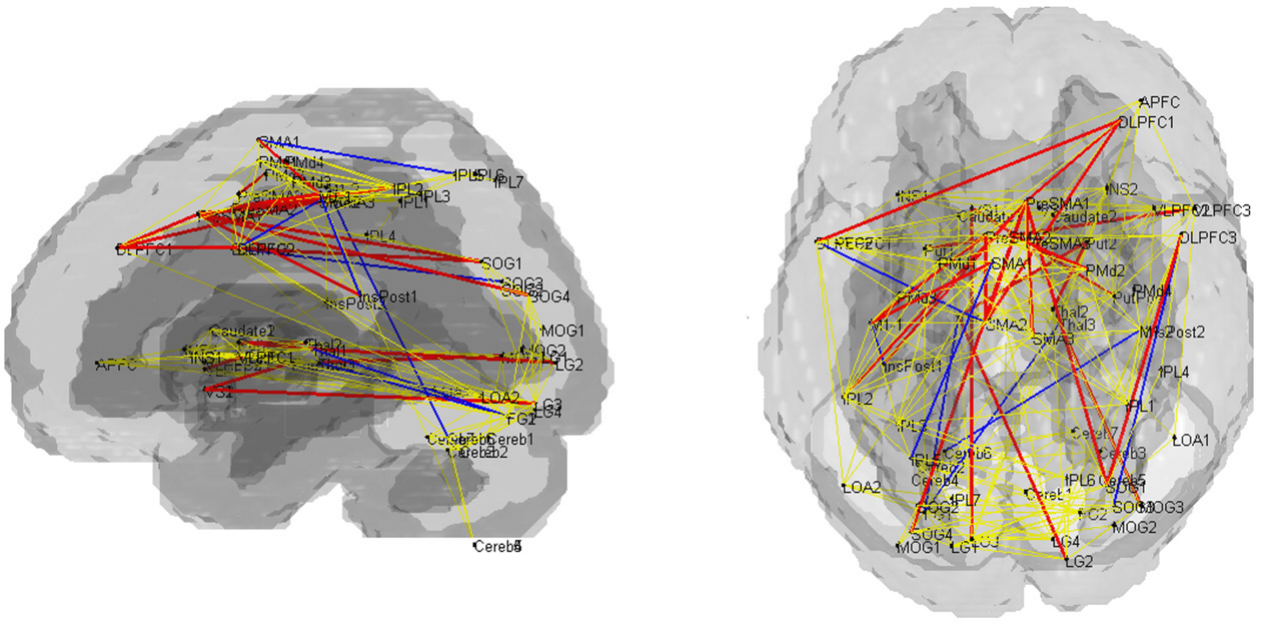
**Pattern of networks in HV, at a wired cost of 0.28**. Yellow lines indicate the edges for which more than half of the subjects in HV and PD groups showed connectivity with cost = 0.28. Blue and red lines indicate the edges with stronger connectivity strength in the HV compared with PD. Red lines indicate edges with significant correlation with the subcortical activity, in the HV and/or PD groups.

Fourteen out of the 27 edges for which connectivity strength was weakened in PD patients were correlated with the subcortical activation during the SI task in HV (Table 4, Figure 3). All the edges that are significantly correlated with subcortical regions are marked with an asterisk in the Table 2. Eighteen of those connection strengths were correlated with the *t*-value of the SI – CTL contrast in the PD patients (Tables 2, 4, Figure 3). Although nine edges overlapped between HV and PD, the correlation patterns were different between the groups. In HV, the correlation between the correlation ratio of the edges and the striatal activation was positive, whereas the correlation between the correlation ratio of the edges and thalamic activation was negative (Table 4). In contrast, in PD, the correlation was positive for the striatum as well as the thalamus. Of note, none of the connectivity was significantly correlated with RT.

**Table 4:** **Correlation between edge strength and subcortical activity**.

**Edges (HV > PD)**		**Subcortical regions**	***r* (normalized)**	***p*-value**
**HV (SUBCORITICAL ACTIVATION)**				
DLPFC1	DLPFC2	PutPost	0.619	0.018
DLPFC3	SOG1	VS2	0.568	0.034
PreSMA1	DLPFC1	VS1	0.563	0.036
PreSMA1	DLPFC1	VS2	0.587	0.027
PreSMA1	DLPFC1	Thal1	−0.694	0.006
PreSMA1	InsPost1	Put2	0.548	0.043
PreSMA1	IPL2	Caudate1	0.645	0.013
PreSMA1	IPL2	Caudate2	0.639	0.014
PreSMA1	IPL2	Thal1	−0.655	0.011
PreSMA1	IPL2	Thal2	−0.601	0.023
PreSMA1	SMA2	Caudate2	0.655	0.011
PreSMA1	SMA2	VS2	0.639	0.014
PreSMA1	SMA2	Thal1	−0.750	0.002
PreSMA1	SMA2	Thal2	−0.721	0.004
PreSMA1	SMA3	VS2	0.753	0.002
PreSMA1	SMA3	Thal2	−0.628	0.016
PreSMA1	SOG1	Thal1	−0.628	0.016
PreSMA2	SMA2	Thal1	−0.576	0.031
PreSMA2	SMA2	Thal2	−0.637	0.014
Thal1	Put2	PutPost	0.690	0.006
Thal1	Put2	Thal1	−0.570	0.033
Thal1	LG2	PutPost	0.569	0.034
Thal2	Put2	PutPost	0.725	0.003
LG3	VS1	PutPost	0.581	0.029
MOG3	Thal3	PutPost	0.544	0.044
**PD (SUBCORITICAL ACTIVATION)**				
PreSMA2	M1-1	Thal2	0.581	0.048
DLPFC3	SOG3	Put1	0.624	0.030
**PD (SI—CTL) (*t*-VALUE)**				
DLPFC1	PreSMA1	Put2	0.698	0.012
DLPFC1	PreSMA1	Thal1	0.583	0.047
DLPFC1	SMA2	VS1	0.632	0.027
DLPFC2	SMA2	Thal1	0.677	0.015
DLPFC2	SMA2	Thal2	0.656	0.021
DLPFC3	SOG1	Caudate2	0.578	0.049
PreSMA1	InsPost1	VS2	0.658	0.020
PreSMA1	IPL2	Put2	0.675	0.016
PreSMA1	IPL2	Thal1	0.664	0.019
PreSMA1	SMA3	Caudate1	0.648	0.023
PreSMA1	SOG1	VS1	0.645	0.024
PreSMA1	SOG4	Put2	0.616	0.033
PreSMA1	SOG4	PutPost	0.584	0.046
PreSMA1	SOG4	Thal1	0.761	0.004
PreSMA1	SOG4	Thal2	0.686	0.014
PreSMA2	M1-1	PutPost	0.676	0.016
PreSMA2	SMA2	PutPost	0.674	0.016
PreSMA2	SMA2	Caudate1	0.717	0.009
PreSMA2	SMA2	Thal1	0.672	0.017
PreSMA2	SMA2	Thal2	0.741	0.006
PreSMA3	PMd2	VS1	0.691	0.013
PreSMA3	PMd2	VS2	0.607	0.036
SMA1	IPL5	VS1	0.696	0.012
SMA1	M1-1	Thal2	0.750	0.005
Put1	VLPFC3	Caudate1	0.629	0.029
Put1	VLPFC3	VS1	0.590	0.043
Put1	VLPFC3	VS2	0.586	0.045
Put2	Thal2	Caudate1	0.689	0.013
PutPost	VS1	Put2	0.700	0.011
PutPost	VS1	VS2	−0.752	0.005
Thal1	LG2	Put2	0.629	0.029
Thal1	LG2	Thal1	0.619	0.032

#### Relationship between correlation ratio of the cortico-subcortical connection and task performance in HV and PD

**Figure 4:**
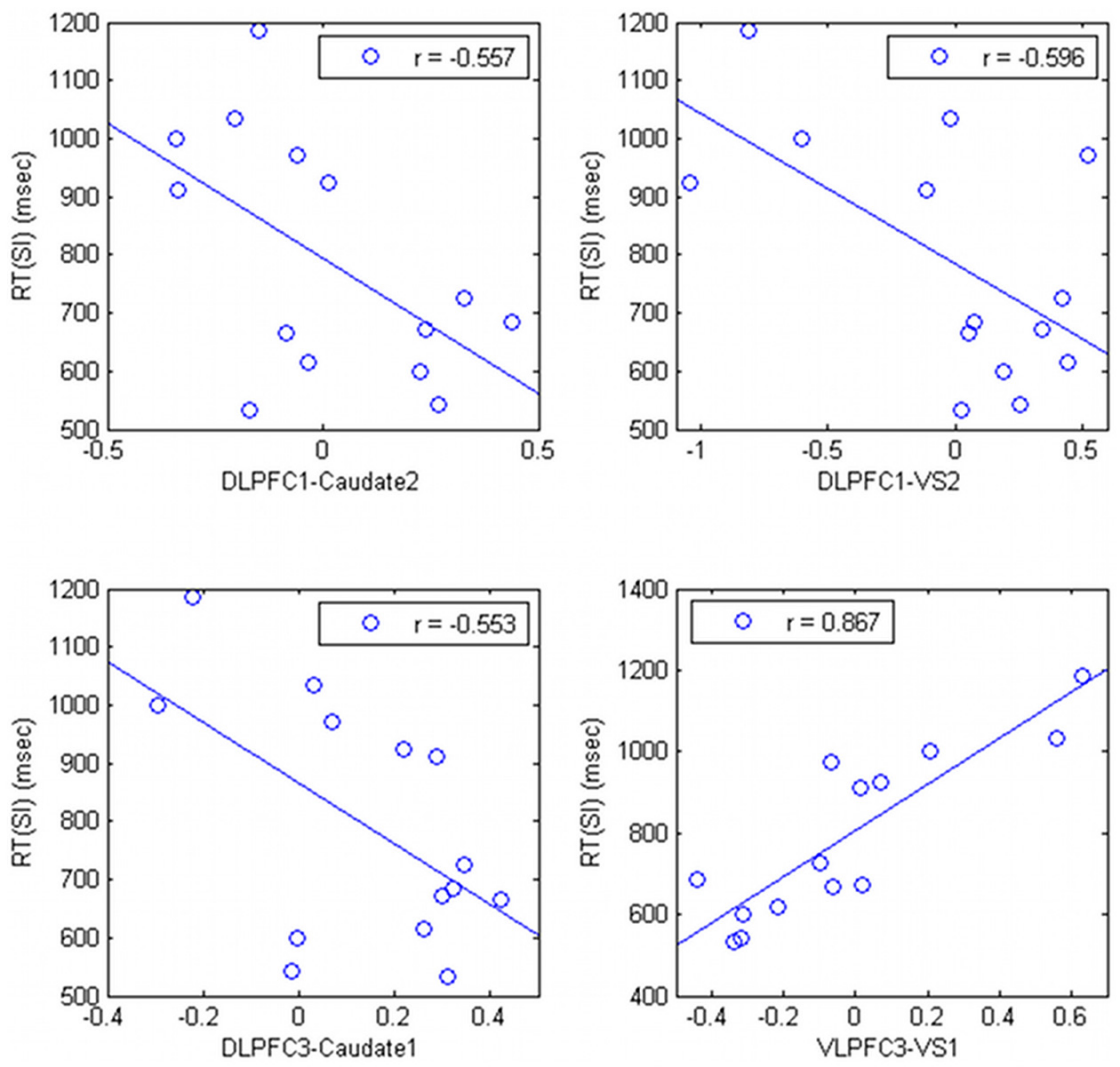
**Relationship between correlation ratio of the cortico-subcortical connections and task performance in HV**.

The connectivity strength between the right DLPFC (DLPFC1 and DLPFC3, in the Table 1) and the striatum was negatively correlated with RT (*r* = −0.557; *p* = 0.038, *r* = −0.596; *p* = 0.245; *r* = −0.553, *p* = 0.040, respectively) (Figure 4). The connectivity strength between right VLPFC (VLPFC3, in the Table 1) and the striatum was strongly positively correlated with RT (*r* = 0.857; uncorrected *p* = 0.00006, corrected *p* = 0.0105).

#### Difference of path length from the primary motor area and other nodes in HV and PD

The path length from the left M1 to the pre-SMA and SMA, left and right DLPFC, and the right posterior putamen, was shorter in the HV compared with PD. The path length from the right M1 to the right insula, right thalamus, right dorsal premotor cortex (PMd), and the left lingual gyrus (LG) was longer in the HV. Results are shown in the Table 5. The mean lengths form the M1-1 to these brain regions as a function of cost are shown in the Complementary data (C-Figure 1).

**Table 5:** **Difference in path length between the primary motor area and other nodes**.

**Nodes**		**Path length (mean ± *SD*)**		***p*-value**
		**HV**	**PD**	
M1-1	SMA1	1.429 ± 0.646	2.083 ± 0.669	0.019
	PreSMA1	1.643 ± 0.633	2.250 ± 0.622	0.027
	PreSMA2	1.429 ± 0.646	2.083 ± 0.669	0.019
	DLPFC1	1.643 ± 0.745	2.333 ± 0.778	0.033
	DLPFC2	1.571 ± 0.646	2.250 ± 0.622	0.016
	PutPost	2.000 ± 0.392	2.417 ± 0.669	0.049
M1-2	INS2	2.357 ± 0.497	1.833 ± 0.577	0.028
	Thal2	2.214 ± 0.579	1.667 ± 0.651	0.037
	PMd3	1.929 ± 0.475	1.417 ± 0.515	0.019
	LG2	2.714 ± 0.469	2.167 ± 0.718	0.040

## Discussion

The BG, modulated by dopaminergic projections (Schultz, 1998; Seamans and Yang, 2004; Joshua et al., 2009; Howe et al., 2013), are thought to play a crucial role in the integration of information from multiple sources in order to make a decision and perform an optimized action (Bar-Gad et al., 2003; Bogacz and Gurney, 2007). The dopaminergic neurons might be involved in the formation of an ideal network combining the cognitive and motor networks in the brain for conducting a series of tasks. We used a graph theory approach on SI finger movements during fMRI acquisition in order to investigate this function in HV and PD patients.

### Graph-theory approach

We used the graph-theory approach while considering wiring cost to select edges relevant for the SI finger movement task used. This could improve the detection of pivotal edges affected by the disease. The peak of the cost efficiency was located at the cost of 0.271 and 0.284 in the HV and PD groups, respectively. Based on a hypothesis that the brains operate optimally with the maximum cost efficiency, maximizing information transfer (Sporns et al., 2000; Achard and Bullmore, 2007), we selected the cost of 0.28 to determine individual networks. Then, the edges included in more than half of the subjects for each group were considered, considering them as a common network for the task. Costs of 0.20 and 0.36 were also calculated, and the results were comparable to the results with a cost of 0.28 (data not shown). Additionally, the results using correlation threshold were also equivalent (patterns of networks with this method are shown in Complementary Data, C-Figure 2), similar to our previous study showing relatively preserved results with the two thresholding methods (Carbonell et al., 2014).

### Cortico-midline connectivity is modulated by BG activity

The main finding of the present study is that connectivity between the mPFC and the motor cortex, extrastriate cortex, and the associative cortex, is weakened in comparison to HV. Furthermore, the strength of the cortico-midline connections was correlated to striatal and thalamic activity. This is in accordance with a previous study that showed a decrease in functional connectivity between the pre-SMA and M1, PMC, IPL, and the cerebellum in PD patients during a SI task (Wu et al., 2011). Our results additionally demonstrate that (1) the lowered connectivity between the mPFC and other cortices remain prominent within the whole brain network, and (2) that the activation in the striatum (the caudate, putamen, and VS) and the thalamus would be involved in supporting this connectivity.

We observed weakened connections between the pre-SMA and M1 in PD patients, and this was accompanied by a reduction of activity in the right posterior putamen, the motor nucleus of the BG (Alexander et al., 1986; Albin et al., 1989). The pre-SMA has anatomical connections with prefrontal areas, without direct connections to the primary motor area (Picard and Strick, 2001). In addition, the pre-SMA is connected to the caudate nucleus, the associative nucleus of the BG, the rostral part of putamen and the globus pallidus (Takada et al., 1998; Lehericy et al., 2004; Akkal et al., 2007). Functionally, the pre-SMA is in a position to influence the motor cortex during action selection (Mars et al., 2009). There may therefore be functional connectivity between the pre-SMA and M1 mediated by the BG system. Interestingly, the mPFC (pre-SMA and SMA) is one of the most frequently reported regions in connectivity analyses (Toro et al., 2008). Moreover, the mPFC, along with other midline structures, is considered to be a structural hub, with dense connectivity to the rest of the brain (Hagmann et al., 2008). A stronger connectivity between the pre-SMA and M1, then, would result in shortening in the path length between M1 and the other cortical regions. In accordance with this hypothesis, we found shorter path lengths between the M1 and the pre-SMA, SMA, putamen, and DLPFC in HV compared with PD patients.

Although striatal and thalamic activity was decreased in PD patients compared with HV (Martinu et al., 2012), the strength of cortico-midline connectivity was positively correlated with activity in the striatum, but negatively correlated with activity in the thalamus in the HV. In contrast, in the PD group, correlations with the cortico-midline connectivity were positive for the striatum as well as the thalamus. The striatum and the thalamus are tightly inter-connected through the cortico-BG-thalamo-cortical loops and the brainstem-thalamo-BG-brainstem loops (Alexander et al., 1986; Albin et al., 1989; McHaffie et al., 2005). Within these circuits, the indirect pathway of the cortico-BG-thalamo-cortical loops exerts opposing effects on the striatum and the thalamus (Alexander et al., 1986; Albin et al., 1989; McHaffie et al., 2005). Therefore, the opposite correlation observed in HV may indicate the involvement of the indirect pathway in the control of cortico-midline connections. Recent animal studies have shown that the direct and indirect pathways are concurrently activated during operant tasks, suggesting that the indirect pathway is positively involved in decision making of actions, rather than simply suppressing unselected actions (Cui et al., 2013; Isomura et al., 2013). Indeed, our observations are in accordance with these recent views.

### Connectivity between the extrastriate visual cortex and subcortical regions

Another important observation in the present study was the decrease in connectivity between the extrastriate visual cortex and the subcortical regions (thalamus and striatum), between the striatum and the thalamus, as well as between the putamen and the VLPFC in the PD group. Our observation suggests that dopamine is involved in controlling the strength of the connectivity. The SI task was designed for internally generated movements. However, the subjects received a visual feedback for pressing a button. Therefore, the task also has a feature of visual-cue-guided goal-directed behavior, showing activation in the visual areas. The extrastriate visual cortex is interconnected with the thalamus at the level of the pulvinar (Jacobson and Marcus, 2007; Leh et al., 2008). In monkeys, this region is connected to the body and tail of the caudate nucleus (Yeterian and Pandya, 1995). The VLPFC is also strongly connected to the striatum, especially in the rostro-ventral part of the caudate nucleus (Leh et al., 2007). Also, the visual cortex also shows functional connectivity with the VLPFC/insula during a visualization task (Ebisch et al., 2013). Nevertheless, the thalamic and striatal regions observed in the present study included the motor components located in the ventrolateral/ventral-posterolateral thalamus and the putamen (Jacobson and Marcus, 2007). Our results suggest, then, a level of integration between subcortical regions involved in visual and motor processes.

### Integration of information between the motor cortex and the prefrontal cortex

Connectivity strength between the superior occipital gyri and the DLPFC showed significant decreases in PD that correlated with subcortical activations. This is in line with a previous study that showed attention-induced fronto-posterior connectivity to be modulated by the BG (van Schouwenburg et al., 2010). The connectivity between the DLPFC and the striatum was not significant (Figure 3), but was negatively correlated to the RT in HV (Figure 4). These observations are in accordance with our previous study, where an increase in temporal connectivity between the DLPFC and the dorsal striatum was associated with shorter RT (Nagano-Saito et al., 2008). It has been suggested that the general role of the DLPFC is to establish a set of responses suitable for a given task (Nathaniel-James and Frith, 2002). Additionally, the DLPFC belongs to the fronto-parietal task control network, which is considered to play a role in the initiation of movement, and in trial-by-trial adaptive control (Dosenbach et al., 2007, 2008). In these respects, the connectivity between the DLPFC and the striatum may correspond to an information flow for trial-by-trial adaptation supported by the large background of the fronto-parietal task control network.

The connectivity between the VLPFC and the striatum was also decreased in PD. This is in accordance with our previous study where we showed that temporal connectivity between the VLPFC and the striatum was reduced when dopamine was depleted (Nagano-Saito et al., 2008). However, the connectivity between the VLPFC and the striatum, as well as the connectivity between the extrastriate visual cortex and the subcortical regions were strong during the task, as opposed to the connectivity between the DLPFC and the striatum (Figure 2). Furthermore, the strength of the connectivity was positively correlated with the individual RT in the HV (Figure 4). We speculated that the stronger connectivity between the VLPFC and the subcortical regions be linked with an increase in visual information flow, which contributes to inhibiting behavioral responses (Sakagami et al., 2001). This may help cognitive function, but requires additional processing for action decision, possibly leading to a longer RT.

Considering the fact that the DLPFC and the VLPFC are endpoints of the ventral and dorsal visual pathways (Sakagami and Pan, 2007), our observation may imply that the subcortical regions play a role in connecting the two visual pathways. This is consistent with a speculation that the BG system would have an important role in the integration of the dorsal and ventral visual pathways' information for decision-making (Cisek and Kalaska, 2010).

The pre-SMA and VLPFC are directly inter-connected (Aron, 2007), and are considered to be a part of the cingulo-opecular task control network involved in set maintenance (Dosenbach et al., 2007, 2008; Power et al., 2011). Although the pre-SMA and VLPFC show synchronized activity during the resting state, we did not observe strong connectivity between them during the SI task (Figure 2), suggesting the two regions have distinct functions. Accordingly, a recent study showed differential modulation of functional connectivity between the VLPFC and the pre-SMA during a reasoning task (Ebisch et al., 2013). More specifically, the VLPFC showed an increase in functional coupling with visual cortices during a visualization task, whereas the pre-SMA displayed an increase in functional coupling with the anterior frontal cortex during an induction task (Ebisch et al., 2013). The pre-SMA and the VLPFC may function for cognitive tasks collaboratively. However, at least in specific tasks, the pre-SMA would allow the establishment of a shorter path between the motor cortex and the other cortical regions, whereas the VLPFC would allow the collection of visual information to the subcortical regions. The striatum and the thalamus, then, may further reinforce these functional connections to generate adequate decisions on resulting actions.

We speculated that mediation of cognitive and motor modules supported by these PFC and subcortical regions in the brain would be important for decision-making processes.

In humans, co-activation of the pre-SMA, VLPFC, DLPFC, along with the thalamus and the striatum has been repeatedly reported during cognitive tasks that require a task-set for visually-guided goal-directed responses (Monchi et al., 2001; Huettel et al., 2004; Chang et al., 2007; Nagano-Saito et al., 2008), and even during our SI tasks (Martinu et al., 2012). Although the exact mechanism behind this integration is unclear, it is most likely mediated by the striato-nigro-striatal connections (Haber, 2003) and the cortico-BG-thalamo-cortical loops (McFarland and Haber, 2002; Calzavara et al., 2007; Draganski et al., 2008).

## Conclusion

**Figure 5:**
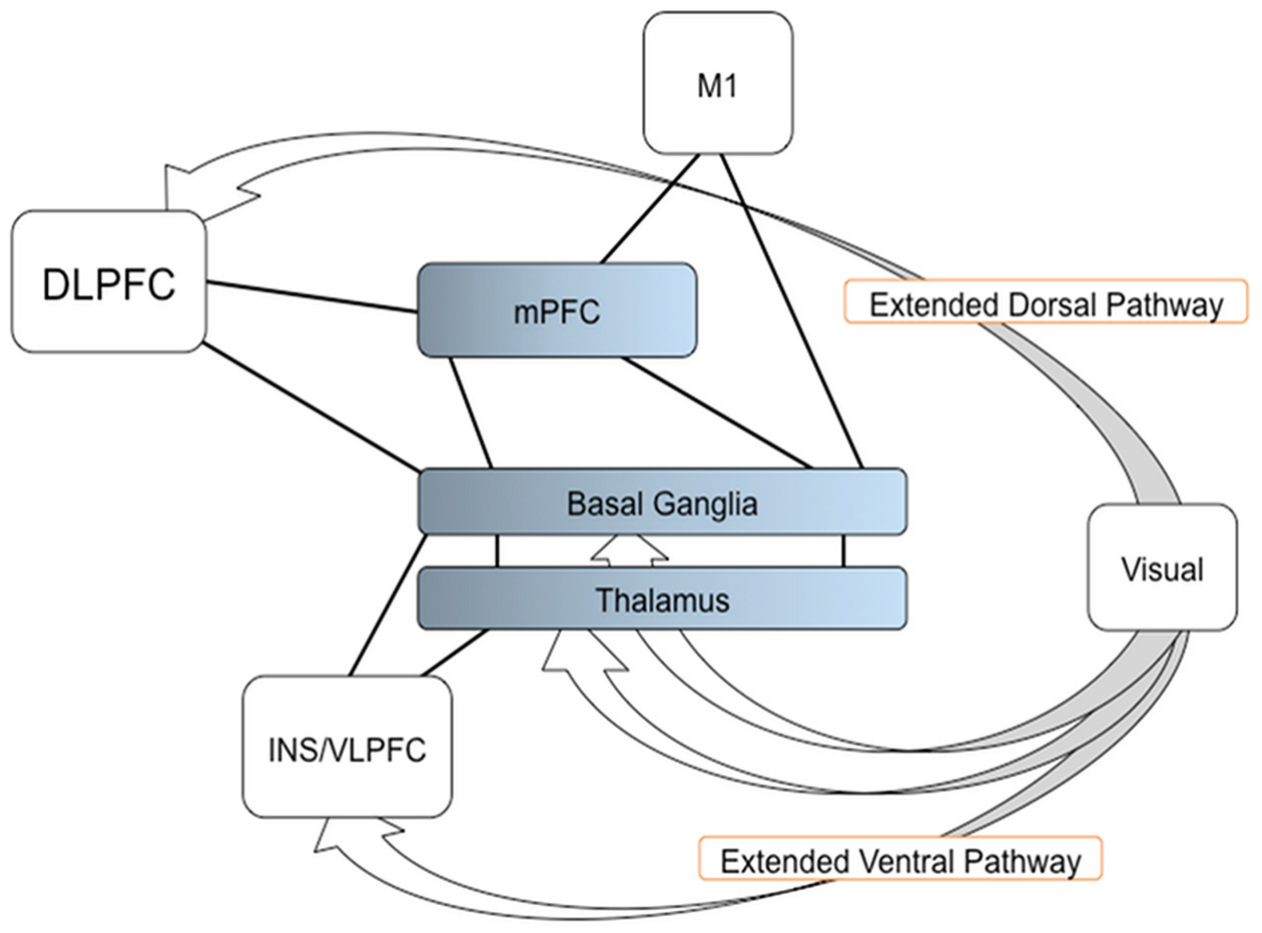
**A model of the BG function for determining a network resulting an action**. The visual information reaches to the frontal regions, such as the DLPFC and VLPFC (endpoints of the extended dorsal and ventral visual pathways). The BG, with the mPFC and thalamus, are involved in the integration of multiple sources of information from the frontal regions and connected to M1, thereby determining a network that leads to the appropriate decision and performance of the resulting action. Arrows indicate the extended dorsal and ventral visual pathways; solid lines indicate the connection shown in current study; Color gradients in the mPFC, BG, and thalamus, indicate transition of cognitive and motor components in the regions.

In the present study, we have applied a graph theory approach to fMRI data. Our results support the hypothesis that through dopaminergic neuro-modulation (Seamans and Yang, 2004; Joshua et al., 2009; Howe et al., 2013), the BG play an important role in the integration of multiple sources of information from functionally specific cortical areas in order to generate an adequate decision on a resulting action (Bar-Gad et al., 2003; Bogacz and Gurney, 2007). More specifically, the BG would integrate information dorsally from parieto-frontal areas, and ventrally from visual areas. Additionally, the BG would allow to shorten the path length between the mPFC and the motor cortex. In networking terms, then, mPFC, DLPFC, and VLPFC interact with the BG to create the adequate network that can combine visual, associative, and motor information to produce the decision on an upcoming action. A possible model for SI task is shown in Figure 5. Additional studies will be necessary in order to determine whether this effect is task-dependent.

### Conflict of interest statement

The authors declare that the research was conducted in the absence of any commercial or financial relationships that could be construed as a potential conflict of interest.

## Abbreviations

BG: basal ganglia
PD: Parkinson's disease
HV: healthy volunteers
SI: self-initiated
ET: externally triggered
CTL: control
pre-SMA: pre-supplemental motor area
SMA: supplemental motor area
M1: primary motor area
mPFC: medial prefrontal cortex
DLPFC: dorsolateral prefrontal cortex
VLPFC: ventrolateral prefrontal cortex
IPL: intra parietal lobe
PMd: dorsal premotor cortex
LG: lingual gyrus
FG: fusiform gyrus
RT: response time
ROI: regions of interest.
