# Supplementary material for: Function of basal ganglia in bridging cognitive and motor modules to perform an action
Source: Front Neurosci. 2014 Jul 8;8:187. doi: 10.3389/fnins.2014.00187 (PMC4086202; doi:10.3389/fnins.2014.00187)

**Complementary data**

The function of basal ganglia in integrating information to perform an action

**Atsuko Nagano-Saito,^1,2^ Kristina Martinu, ^1^ Oury Monchi^1,2^**

**^1^** Centre de Recherche, Institut universitaire de Gériatrie de Montréal, 4545 Queen Mary, Montréal, QC, H3W 1W5, Canada

**^2^** Department of Radiology, Université de Montréal, 2900, boul. Édouard-Montpetit Montréal, QC, H3T 1J4, Canada

**Corresponding author: Dr. Atsuko Nagano-Saito**

**Phone: 1-514-340-2800 ext 3590**

**E-mail address:** [atsuko.nagano@gmail.com](mailto:atsuko.nagano@gmail.com)

**
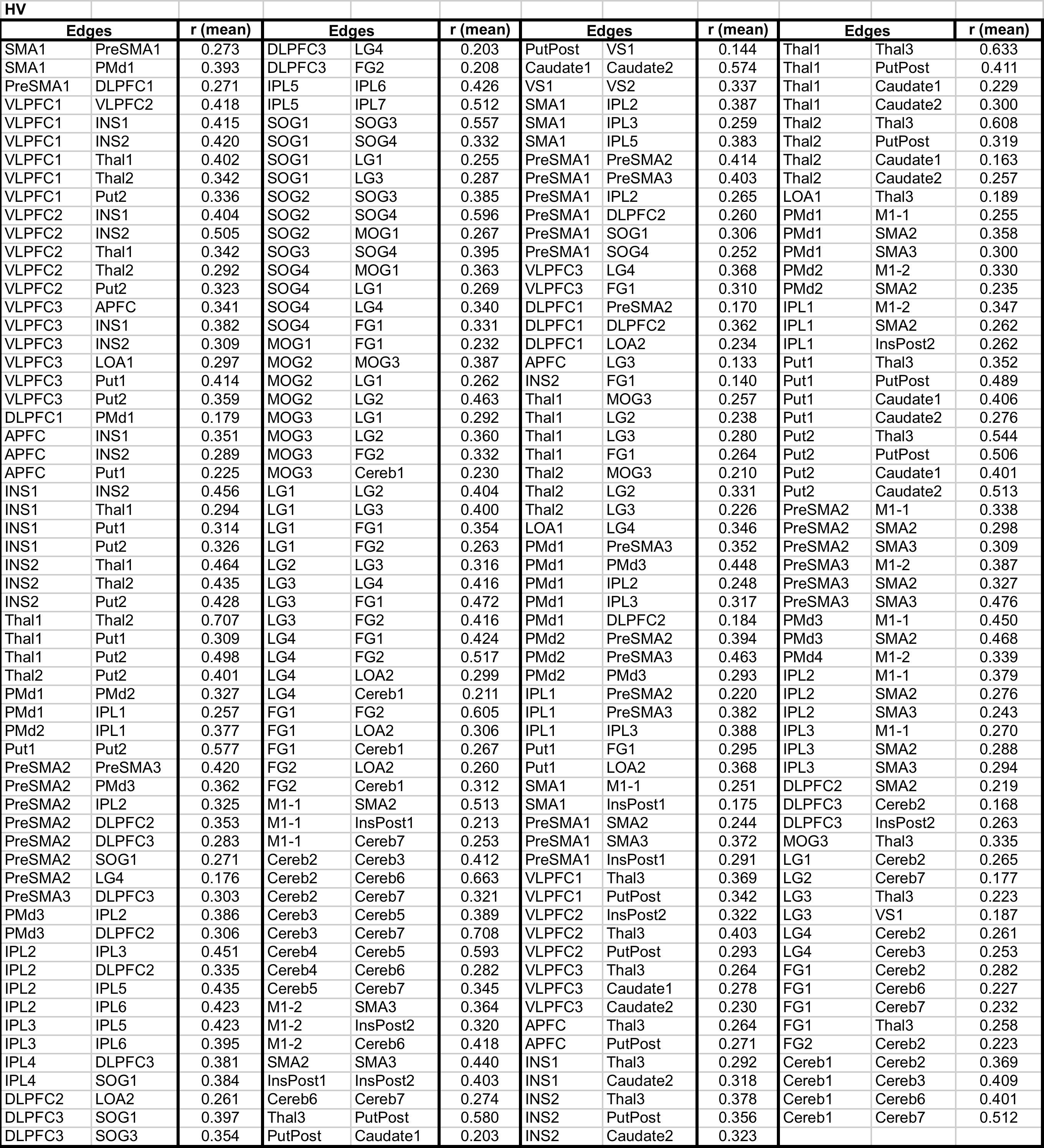
C-Table 1. All the connectivities in the HV and PD, respectively (cost = 0.28)**


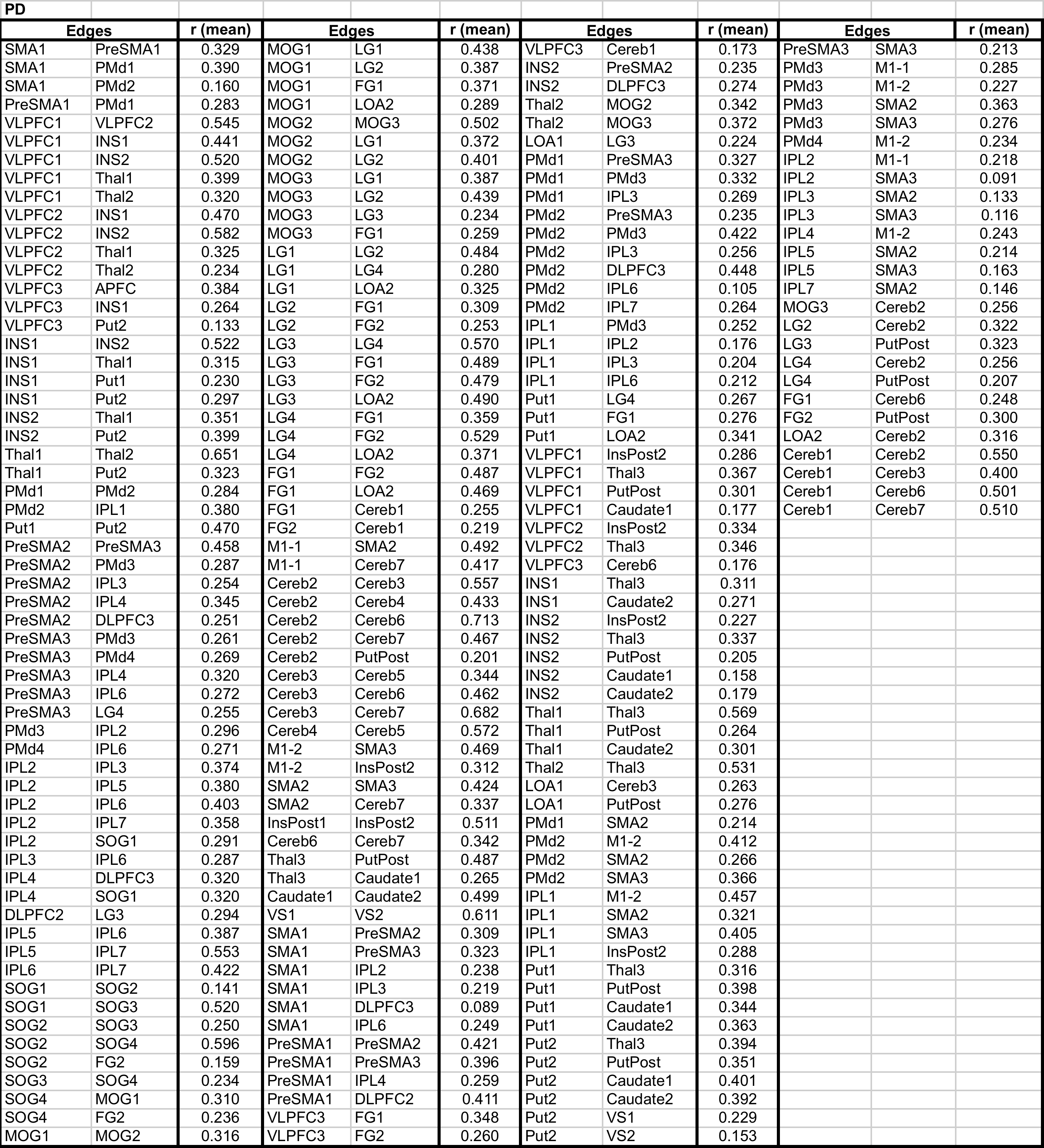


**C-Figure 1. Mean path length from the M1-1 in the HV and PD, as a function of cost**


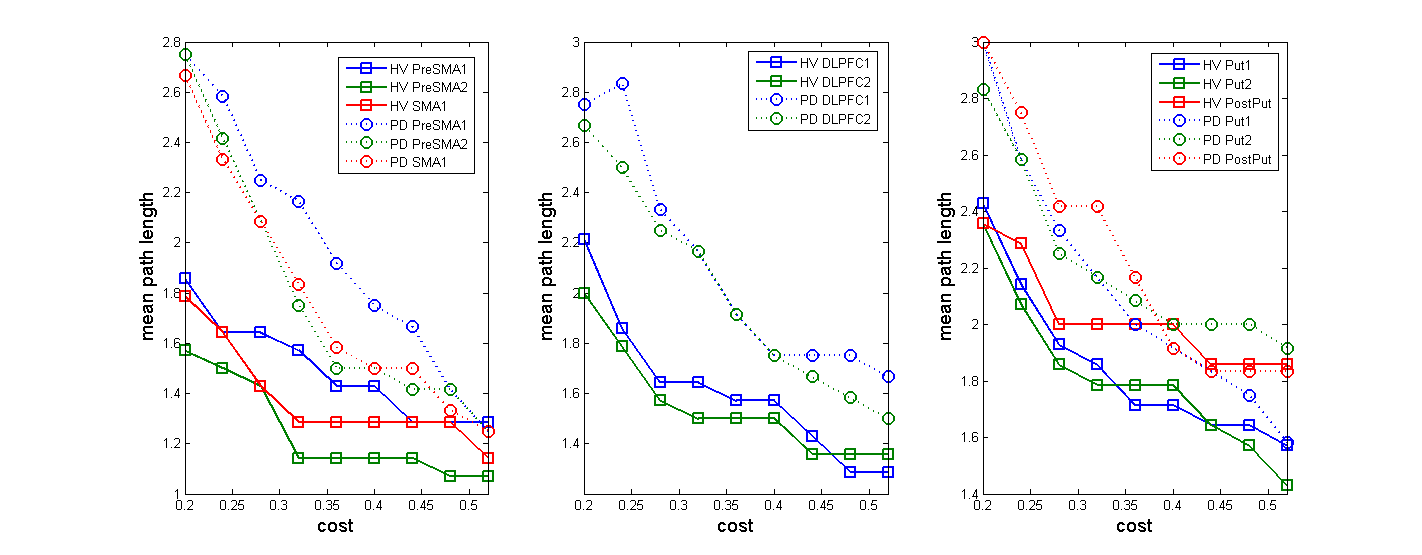


The difference of the path length between the groups became smaller when the cost increased, preserving the tendency shown in Table 5. At the cost = 0.52, the path length between the M1-1 and the PreSMA2 was still significantly shorter in the HV, compared with the PD. However, the path length between the M1-1 and the PostPut did not show any difference. Instead, the path length between the M1-1 and the Put2 was significantly shorter in the HV. The correlation threshold at the cost 0.52 was 0.114 ± 0.0750, and 0.0939 ± 0.0617, in the HV and the PD, respectively.

**C-Figure 2 Pattern of networks in HV and PD, with correlation threshold method**

Lines indicate the edges for which the mean correlation ratio in each group showed more than the correlation threshold. The numbers in the figure indicate the correlation threshold. Yellow lines indicate the edges observed in both the HV and PD. Blue lines indicate the edges observed in the HV. Red lines indicate the edges observed in the PD.


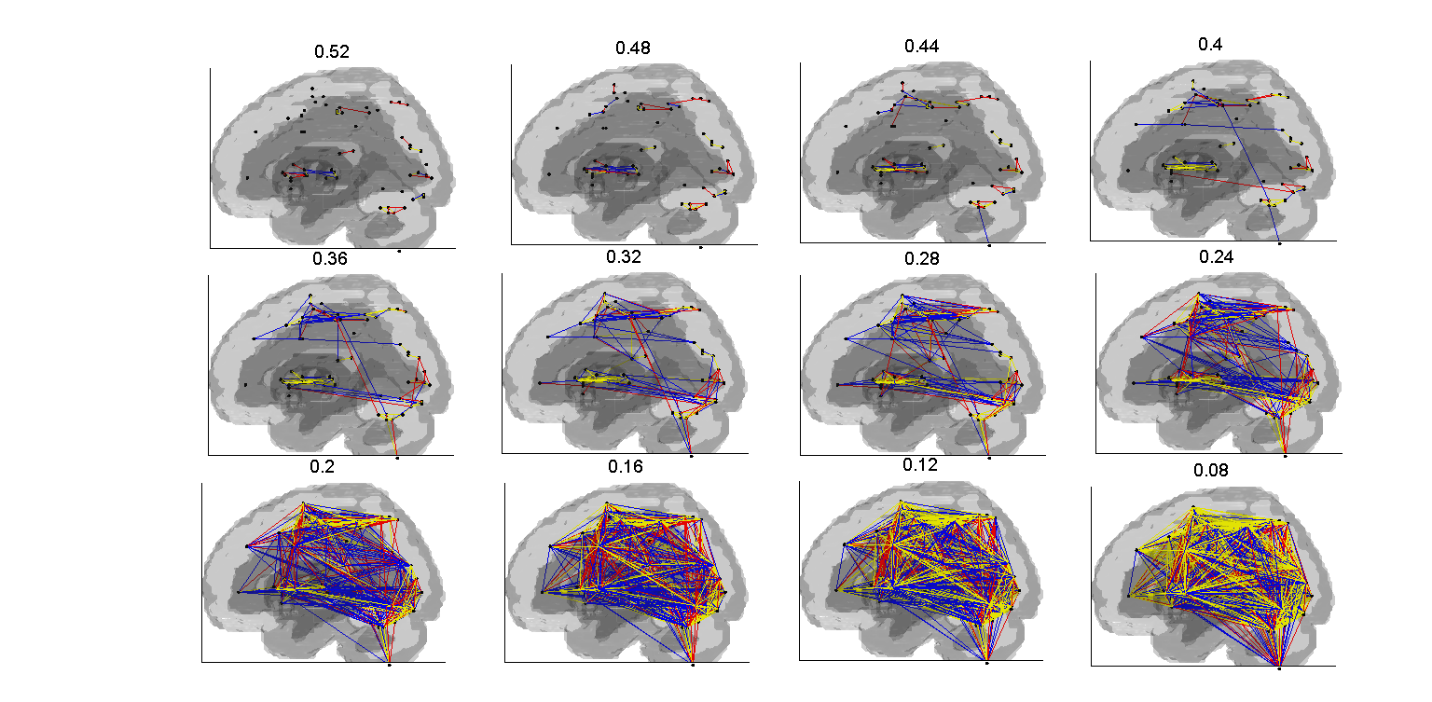

Supplement: Supplementary file 1 [file DataSheet1.DOCX]
